# Assessment of the Physicochemical and Conformational Changes of Ultrasound-Driven Proteins Extracted from Soybean Okara Byproduct

**DOI:** 10.3390/foods10030562

**Published:** 2021-03-08

**Authors:** Gilda Aiello, Raffaele Pugliese, Lukas Rueller, Carlotta Bollati, Martina Bartolomei, Yuchen Li, Josef Robert, Anna Arnoldi, Carmen Lammi

**Affiliations:** 1 Department of Human Science and Quality of Life Promotion, Telematic University San Raffaele, 00166 Rome, Italy; gilda.aiello@unimi.it; 2 NeMO Lab., ASST Grande Ospedale Metropolitano Niguarda, 20133 Milan, Italy; raffaele.pugliese@nemolab.it; 3 Fraunhofer Institute for Environmental, Safety and Energy Technology UMSICHT, 46047 Oberhausen, Germany; Lukas.Rueller@umsicht.fraunhofer.de (L.R.); josef.robert@umsicht.fraunhofer.de (J.R.); 4 Department of Pharmaceutical Sciences, University of Milan, 20133 Milan, Italy; carlotta.bollati@unimi.it (C.B.); martina.bartolomei@unimi.it (M.B.); yuchen.li@unimi.it (Y.L.); anna.arnoldi@unimi.it (A.A.)

**Keywords:** atomic force microscope, circular dichroism, phytic acid, green extraction, soybean proteins, soybean okara

## Abstract

This study was aimed at the valorization of the okara byproduct deriving form soy food manufacturing, by using ultrasound at different temperatures for extracting the residual proteins. The physicochemical and conformational changes of the extracted proteins were investigated in order to optimize the procedure. Increasing the temperature from 20 up to 80 °C greatly enhanced the yields and the protein solubility without affecting the viscosity. The protein secondary and tertiary structures were also gradually modified in a significant way. After the ultrasonication at the highest temperature, a significant morphological transition from well-defined single round structures to highly aggregated ones was observed, which was confirmed by measuring the water contact angles and wettability. After the ultrasound process, the improvement of peptides generation and the different amino acid exposition within the protein led to an increase of the antioxidant properties. The integrated strategy applied in this study allows to foster the okara protein obtained after ultrasound extraction as valuable materials for new applications.

## 1. Introduction

Soybean (*Glycine max*) is a protein-rich oilseed widely employed in the food industry for producing soy foods and beverages. Thanks to its nutrient content, soybean is used in several dishes as a valid alternative to meat, and it is added in various vegan-friendly food and beverages [1]. Soybean stands out not only for its nutritional value but also for the health benefits it provides (i.e., lowering of blood cholesterol level, increasing of bone density, and minimization of the risk of cancer development) [2]. 

The rising demand for plant-based foods is strengthening the growth of the soy food market across the globe. In fact, the global soy food market was worth 38.7 billion US$ in 2018 and is expected to reach the value of 53.1 billion US$ by 2024, registering a compound annual growth rate (CAGR) of around 5% during 2019–2024. In general, soybeans with a high protein content are chosen for the preparation of soymilk, compared to those utilized for oil extraction [3]. 

During soymilk and tofu production, soybeans are milled under hot (>80 °C) and alkaline (pH 8.0) conditions to guarantee a protein solubilization as well as to inactivate trypsin inhibitors and the enzyme lipoxygenase [4]. Insoluble materials are removed from the slurry using centrifugation: this process results in the production of a soy base, which is the precursor of soymilk or tofu, and a solid by-product generally named with the Japanese word okara, which contains about 50% dietary fibers, 25% protein, 10% lipid, and other nutrients [5,6] and is generally discarded or used as feed ingredient. Due to this interesting composition, okara byproducts have been already assessed for the extraction of fibers [7] and polysaccharides [8] as well as for the manufacture of snack [9]. Although it would be certainly advisable to extract the residual proteins for human use, this is impaired by the okara structure. A specific sustainable protein extraction methodology is thus required to reach this goal. 

The ultrasound technology has been widely studied in the food industry for aiding the extraction of components of interest from plant starting materials [10,11,12]. The ultrasound technology shows promise as a green extraction technology, reasons including reductions in extraction times, less and more sustainable solvent use, and more effective energy utilization, as well as improvement of the quality of the product [13,14]. The success of ultrasound is attributed to the cavitation phenomenon. In fact, upon asymmetric bubble collapse, liquid jets are formed that can disrupt cells upon contact with cell walls, [15] causing the release of intracellular compounds. 

Within the storage cells of soybean, protein is organized in 5–20 μm protein bodies, surrounded by a cytoplasmic network containing oil bodies in the size range of 0.2–0.5 μm stabilized by proteinaceous oleosins [16]. 

Different methods are applied for the protein extraction of soybean and okara. More specifically, the extraction of proteins is carried out using acid and alkaline conditions [17,18], enzyme assisted extractions [19,20], and more recently with the aid of ultrasonication technology [13], which is nevertheless still rarely employed. The complexity of soybean cellular microstructure influences the protein extraction in normal conditions, but thanks to the cavitation phenomenon, an improvement of extraction yields from hexane-defatted soy flakes is achievable at lab-scale. Protein functionality improvement from soybean protein isolate and concentrates has also been reported with positive results in protein solubility and particle size reduction [21]. In addition, the ultrasound treatment significantly increases the solubility of isolated soy proteins in water [22].

In light with these observations, this study is aimed at assessing the physicochemical and conformational changes of ultrasound-driven extracted proteins from soybean okara processing materials. To achieve this goal, the protein extraction was carried out using ultrasound at 20, 60, and 80 °C obtaining the samples named SoK_U20, SoK_U60, and SoK_U80, respectively. The high temperature limit was chosen taking into account that temperatures up to 80 °C are used in the processes where okara is produced as byproduct. The assessment of the changes in the protein profile, concentration, secondary and tertiary structure, hydrophobicity, solubility, antioxidant capacity, and rheological and morphological features was performed in comparison with untreated soy okara protein (SoK_nU). Herein, a combination of different techniques and an integrated strategy were applied in order to foster the okara protein samples obtained after ultrasound extraction as valuable and high-quality products for new applications and to provide, therefore, a more sustainable way to solve the environmental criticism related to the huge quantities of okara produced annually, which pose a significant disposal problem.

## 2. Materials and Methods

### 2.1. Chemicals and Reagents

All chemicals and reagents were of analytical grade and commercially available. More details are reported in Supplementary Information.

### 2.2. Samples and Ultrasonic System

The experimental investigations were performed with an ultrasonic batch system (TC 10, BSONIC GmbH, Germany) that allows power inputs of up to 4 kW at a frequency of 18 kHz and an oscillation amplitude of 45–60 µm. The probe tip has a diameter of 41.75 mm. Regulation of power input and recording of process parameters is done via PC. Berief Food GmbH, Germany, supplies the soy okara samples as frozen 3 kg units directly from the production process. For the liquid mixture of soy okara, tap water is used.

### 2.3. Ultrasound-Assisted Processing of Okara

Experiments of ultrasound-assisted extraction of proteins were performed at Fraunhofer UMSICHT, Germany, using the materials and ultrasonic system mentioned above. A defined ratio of 1:2.5 of soy okara and water was heated up to the experimental temperature of 20 °C (SoK_U20), 60 °C (SoK_U60), and 80 °C (SoK_U80), respectively, with continuous stirring. The ratio of 1:2.5 has been defined in previous experimental investigations, which are part of a separate publication. This ratio was selected as optimum concerning an effective protein extraction, the disintegration of okara, and the minimization of total treatment volume. The pH of the mixture was 6.5 (±0.14) at 20 °C. The ultrasound treatment was controlled and measured via PC and was conducted by immerging the ultrasound sonotrode into the beaker containing the samples (5 L). Ultrasound parameters of power input (4 kW), correlating oscillation amplitude (µm), and the treatment time of 0.5 min were fixed. Hence, the total energy input for all samples was 24 kJ/ L. These ultrasound treatment parameters have been defined ahead as part of a separate publication showing that they allow the best protein extraction yield. Directly after the ultrasound treatment, the processed samples were centrifuged at 12,298 *g* for 15 min (Avanti JXN-26, Beckmann Coulter) to separate solid and liquid fractions. Both fractions were separately freeze-dried (Alpha 2–4 LSC plus, Christ) in order to allow a simpler storage and delivery of the samples. The protein extraction yields were determined by Kjeldahl analysis.

### 2.4. Soy Okara Protein Extraction

Proteins from sonicated okara were extracted by modifying a method previously described [23]. More details are reported in Supplementary Materials.

### 2.5. Molecular Weight Distribution and MS Analysis

The molecular weight distribution of untreated and sonicated okara proteins was determined using reducing dodecyl sulfate-polyacrylamide gel electrophoresis (SDS-PAGE). The protein samples were prepared by mixing 15 μL of each sample with 10 μL of Laemmli buffer (4% SDS, 20% glycerol, 10%, 0.004% bromophenol blue, and 0.125 M Tris–HCl, pH 6.8). The mixtures were boiled for 5 min at 95 °C, and 25 μL of the mixture was loaded into each lane. The gel was composed of a 4% polyacrylamide stacking gel over a 12% resolving polyacrylamide gel. The electrophoresis was conducted at 100 V until the dye front reached the gel bottom. Staining was performed with colloidal Coomassie Blue and destaining with 7% (*v/v*) acetic acid in water. The gel image was acquired by using the Bio-Rad GS800 densitometer and analyzed by using the software quantity One 1-D. Gel bands for the SoK_U20, SoK_U60, and SoK_U80 lane were sliced, digested with trypsin according to a preceding paper, [23] and analyzed by nano-HPLC-CHIP-ESI Ion Trap using the same experimental conditions previously reported [24]. The MS data were analyzed by Spectrum Mill Proteomics Workbench (Rev B.04.00, Agilent), consulting the *Glycine max* (251326 entries) protein sequences database downloaded from the National Center for Biotechnology Information (NCBI) [25]. For MS/MS search the oxidation of methionine residues was set as variable modifications.

### 2.6. Peptidomic Profiles

The peptidomic profiles of the samples were obtained after ultrafiltration through 3 kDa molecular weight cut-off membranes (Amicon^®^ Ultra, Millipore, Billerica, MA, USA). The recovered peptides were analyzed by nano LC–MS/MS analysis according to the chromatographic and MS condition reported in the material and methods. Figure S1 shows the MSn TIC of Sok_U80, Sok_U60, and Sok_U20. The MS data were analyzed by Spectrum Mill Proteomics Workbench (Rev B.04.00, Agilent), consulting the *Glycine max* (251326 entries) protein sequences database downloaded from the National Center for Biotechnology Information (NCBI). For MS/MS analysis and searching against a polypeptide sequence database, a non-enzyme specific search considering all of the possible proteolytic cleavages was selected as criteria.

### 2.7. Protein Solubility and Water Binding Capacity

Protein solubility water binding capacity (WBC) were assessed according to a literature method with slight modifications [26]. More details are reported in Supplementary Materials. 

### 2.8. Free Sulfhydryl Group Determination

Each sonicated sample (1 g) was dispersed into 9 mL deionized water to obtain the protein solution (1 wt%). Protein solutions were stirred for 2 h and centrifuged at 11,200 *g* for 20 min at 4 °C. The protein concentration in the samples before centrifugation and in the supernatants after centrifugation was determined according to the Bradford assay using BSA as a standard. The protein solubility was expressed as grams of soluble protein per 100 g of protein. All determinations were conducted in triplicate.

The water binding capacity (WBC) was assessed according to a literature method with slight modifications [26]. Briefly, 1 g of sample was dispersed in 10 mL distilled water in a 15 mL pre-weighed centrifuge tube. The dispersions were stirred for 30 min and then centrifuged at 7000 *g* for 25 min at room temperature. The supernatant was discarded, and the tubes were weighed to determine the amount of retained water per gram of sample.

### 2.9. Intrinsic Fluorescence Spectroscopy

The intrinsic fluorescence spectrum of each sample was obtained using a fluorescence spectrophotometer (Synergy H1, Biotek, Bad Friedrichshall, Germany). The samples were diluted in phosphate-buffered saline (PBS, 10 mM, pH 7.0) in order to reach the equal concentration of 0.05 mg/mL and transferred in Greiner UV-Star^®^ 96 well plates flat bottom clear cyclic olefin copolymer (COC) wells (cycloolefine). The excitation wavelength was set as 280 nm, while the excitation and emission slit widths were each set as 5 nm. The emission wavelength range was set up from 300 to 450 nm, and the scanning speed was 10 nm/s.

### 2.10. Circular Dichroism (CD) Spectroscopy

CD spectra were recorded in continuous scanning mode (190–300 nm) at 25 °C using Jasco J-810 (Jasco Corp., Tokyo, Japan) spectropolarimeter. Following protocols reported in Supplementary Materials. The estimation of the peptide secondary structure was achieved by using a literature method [27].

### 2.11. Water Contact Angle Measurements

Contact angle measurements were performed on a Krüss Easy Drop instrument using freshly distilled water passed through a MilliQ apparatus. Sample powders were deposited on glass slides according to a literature procedure [28]. A 8 μL drop was produced and placed on the surface. Videos with 25 fps resolution were recorded. For each sample 2 to 3 measurements were performed, determining the first measurable contact angle and the total time needed for the droplet complete absorption.

### 2.12. Rheological Test

The rheological properties of soy okara proteins were tested using a stress/rate-controlled Kinexus DSR Rheometer (Netzsch) mounted with a parallel plate geometry (acrylic diameter, 20 mm; gap, 34 μm). All measurements were performed at a controlled temperature of 25 °C. The viscosity of okara proteins was measured using a flow step program, at increasing shear rate (0.01–1000 s^−1^), to evaluate their non-Newtonian behavior. Afterwards, to evaluate the storage (G’) and loss moduli (G’’), frequency sweep experiments were recorded at 0.1–100 Hz (strain 0.1%, in LVR). Each experiment was performed in triplicate. Data were processed using Origin^TM^ 8 software (Northampton, MA, US).

### 2.13. Atomic Force Microscopy (AFM)

AFM measurements were captured in tapping mode by using a Tosca system (Anton Paar) using single-beam silicon cantilever probes (Bruker RFESP–75 0.01–0.025 Ohm-cm Antimony (n) doped Si, cantilever f0, resonance frequency 75 kHz, constant force 3 N m^−1^). AFM images were taken by depositing 3 μL solutions (final concentration of 0.1 mg mL^−1^) onto freshly cleaved mica. The samples were kept on the mica for 5 min; subsequently, they were rinsed with distilled water to remove loosely bound peptides and then dried under ambient conditions for 24 h. The AFM morphological parameters were obtained using the Matlab-based open-source software FiberApp (Schmelzbergstrasse, Zurich).

### 2.14. Scanning Electron Microscopy (SEM)

Scanning electron microscopy (SEM) was performed with a Vega-3 microscope from TESCAN GmbH, Germany, at 20 kV acceleration voltage under high vacuum. To guarantee a high resolution, the images were taken with a secondary-electron detector (SE). Freeze-dried samples were fixed on a special two-sided adhesive tape and coated by a 10 nm gold surface in order to prevent electrostatic charging. 

### 2.15. Determination of the Scavenging Activity by the DPPH Assay

The DPPH assay to determine the antioxidant activity in vitro was performed by a standard method with slight modifications [29]. Detailed information is reported in Supplementary Materials.

### 2.16. Phytic Acid (PA) Determination

Lyophilized samples were used for phytic acid determination, following the modified colorimetric method [30]. Aqueous phytic acid standards in concentrations of 0–100 μg/mL were used for quantification. Aliquots of 100 μL of samples and standards were diluted 25 times with 2.4 mL of H_2_O; 600 μL of the diluted samples and standards were combined with 200 μL of modified Wade reagent (0.03% of FeCl_3_6H_2_O and 0.3% of sulfosalicylic acid), and the absorbance was measured at 500 nm.

### 2.17. Statistical Analysis

Data are presented as mean ± s.d. using GraphPad Prism 8 (GraphPad, La Jolla, CA, USA). Statistical analyses were carried out by t student test and ANOVA. *p*-values < 0.05 were considered significant.

## 3. Results and Discussions

### 3.1. Effect of Ultrasound Treatments on the Morphology of Soy Cells in Okara

Proteins are largely responsible for the main features of most foods, since their composition influences nutritional, rheological, and sensory properties. The new process may induce chemical modifications in the protein, impacting on the nutritional and technological features of the final products. The experimental setup used for the ultrasound-assisted processing of okara is illustrated in (Figure S1), while the parameters for the production of the samples are reported in (Table 1). Low ultrasound frequency at 18 kHz was used in order to guarantee an effective acoustic cavitation. In case of low frequencies, the periods of positive and negative pressure changes within the mechanical ultrasound wave are longer. Therefore, the growth process of the cavitation bubble is more effective, and higher bubble diameters can occur. Thus, the bubble implosion takes place with higher forces and results in a more disruptive cell disintegration in comparison to higher frequencies [13]. Accordingly, the oscillation amplitude is responsible for the net pressure change within the sonicated liquid. With higher amplitude, the pressure difference is higher, and acoustic cavitation bubbles are likely to increase in size. Therefore, a frequency of 18–22 kHz at a maximum oscillation amplitude is favorable for ultrasound disintegration and defines the ultrasound power input. Energy input is the product of power input and treatment time in relation to the sonicated volume. High energy input correlates with a high rate of disintegrated cells. However, treatment time should be minimized in order be able to convert the disintegration process to an industrial scale. Previous investigations that are part of a separate publication have shown that the selected ultrasound conditions guarantee an optimum of protein extraction and feasibility for potential industrial scale-up. 

**Figure 1:**
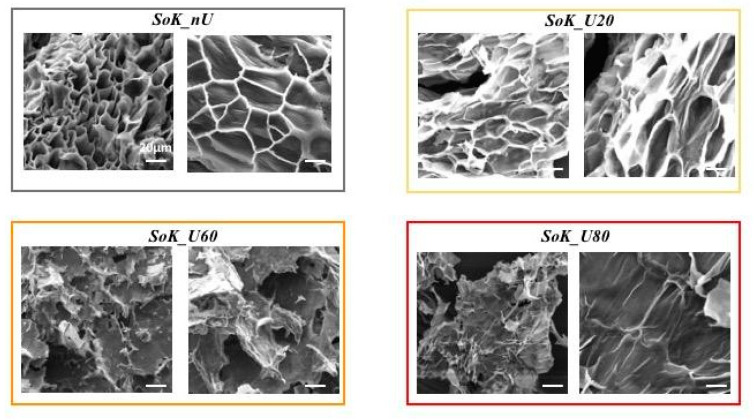
SEM of protein okara obtained with and without the ultrasound treatments. *Scheme 1600*x, r.: 4000x), Sok_U20 (ultrasonicated at 20 °C, magn.: l.: 800x, r.: 1600x), Sok_U60 (ultrasonicated at 60 °C, magn.: l.: 160x, r.: 400x) and Sok_U80 (ultrasonicated at 80 °C, magn.: l.: 400x, r.: 1600x).

In order to evaluate the effect of ultrasonication coupled to the temperature gradient on the structure and morphology of soybean cells, scanning electron microscope analysis was carried out. Figure 1 shows that there are clear differences among the structures of the untreated and ultrasound treated samples. While both disrupted and intact cells co-exist in the sample treated with ultrasound at 20 °C (SoK_U20), the treatment at 60 °C (SoK_U60) and 80 °C (SoK_U80) produces visible disruptions of the cell structures. Since palisade-like cell structures contain a significant amount of protein bodies, an effective disruption of cells is indispensable for an efficient protein recovery (the protein extraction yields from SoK_U80, SoK_U60, and SoK_U20 were 23.5%, 14.9%, and 10.2%, respectively).

### 3.2. Effect of Ultrasound Treatments on the Molecular Weight Distribution of Extracted Proteins

**Figure 2:**
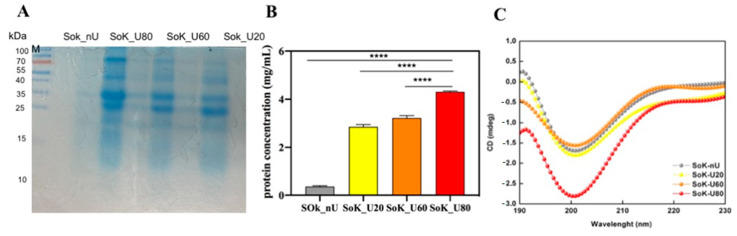
Protein profile, concentration, and CD spectra. (**A**) Reduced SDS-PAGE protein profile of ultrasonicated and untreated proteins (M, pre-stained molecular marker). (**B**) Determination of the protein concentration by the Bradford assay. (**C**) CD spectra of okara proteins. SoK_nU (untreated), Sok_U20 (ultrasonicated at 20 °C), Sok_U60 (ultrasonicated at 60 °C), and Sok_U80 (ultrasonicated at 80 °C).

The effects of ultrasound treatments on the molecular properties of extracted proteins were explored by evaluating their molecular weight profile using reducing electrophoresis. Figure 2A shows the SDS-PAGE profile of the untreated and sonicated protein samples. Under reducing conditions, four intense bands were observed for all the samples, with molecular weight ranges of 70–100 kDa, 40–55 kDa, 25–30 kDa, and ~18 kDa, respectively. The identified proteins for each band are reported in Table S1. Specifically, alpha and beta-subunits of conglycinin were identified at 70 kDa and 50 kDa, respectively. These findings are in line with those reported by other authors according to which no changes in the molecular weight profiles of squid mantle proteins, [31] walnut protein isolate [32], and soybean proteins were observed after sonication [33]. 

The intensity of the electrophoretic bands of sonicated samples at different temperature was greater than that of the untreated sample, which may be attributed to the greater water-solubility of the sonicated proteins. This evidence was confirmed also by Bradford assay according to which the detected amounts of protein were 4.31 ± 0.03, 3.22 ± 0.01, 2.85 ± 0.1, and 0.36 ± 0.04 mg/mL for SoK_80, SoK_60, SoK_20, and SoK_nU, respectively. Therefore, the ultrasound process performed at room temperature (SoK_U20) led to an improvement of protein extraction yield by up to 6.9-fold versus SoK_nU (Figure 2B). This is in agreement with other studies that have shown that ultrasound improves protein extraction yield from soybeans in a lab-scale system [34].

The study on the impact of the temperature on the ultrasound aided protein extraction indicated that in SoK_U60 and SoK_U80, the protein extraction yields were increased by 13.0% and 51.2%, respectively, vs SoK_U20. This high recovery yield was in agreement with SEM investigation that highlighted the progressive destruction of the cell structure in ultrasound treated samples as a function of the temperature leading to an improvement of released proteins. 

After 3 kDa cut-off filtration, each sample was submitted to a peptidomic investigation. The MS/MS analysis revealed that the ultrasonication induced a progressively greater peptide release that proportional to the increasing temperature (Figure S2). In agreement with the degree of hydrolysis (data not shown), the peptide sequences identified after ultrasound treatments increased also as a function of the temperature (Table S2): in fact, 24, 30, and 37 different peptides were identified in Sok_U20, Sok_U60, and Sok_U80 samples, respectively. Interestingly, in all samples the peptide lengths were similar, ranging from 6 to 29 amino acid residues, and the pI values were comprised between 3.8 and 9.9.

### 3.3. Circular Dichroism (CD) of Okara Proteins

To investigate the effect of ultrasound treatment and temperature combination on the secondary structure of extracted proteins, CD spectra in the far UV region of 190–230 were recorded (Figure 2C). One positive and one negative Cotton effect at 193 and 200 nm, respectively, were observed for SoK_nU, suggesting an α-helix rich conformation. Increasing the temperature of the ultrasound treatment from 20 and 60 °C, the intensity of the Cotton effect peaks was greatly decreased, suggesting a structural transition from α-helix into β-sheet rich conformations. The latter conformation was clearly visible in the SoK_U80 sample, where one maximum peak at 195 nm and one minimum peak at 200 nm were observed, demonstrating a redshift of the maximum peak, thus indicating a highly ordered β-sheet rich structures after heating the sample at 80 °C (Figure 2C). 

To gain further information about the secondary structure of soybean okara proteins, the Raussens and coworkers’ tool was applied [27]. Results, summarized in (Table 2), suggest that a reduction of the percentage of α-helices and an improvement of β-sheet were obtained for the proteins after ultrasound treatment (Sok_U20) versus the untreated sample (SoK_nU). Clearly the increase of temperature coupled to ultrasonication induced a significant secondary structure variation. Overall, reductions of α-helices up to 11.3% and 1.1% at 60 and 80 °C, respectively, vs SoK_U20 (26.7%) were observed. In addition, increases of β-sheet up to 33.2% and 37.7% at 60 and 80 °C, respectively, vs SoK_U20 (28.1%) were observed. An overall slight improvement of random coil was also observed for each experimental condition.

### 3.4. Free-Sulfhydryl Group (SH) Content

Sulfhydryl groups (SH) and disulfide bonds (S-S) are important chemical bonds that stabilize the conformation of protein molecules and play very important roles in functional properties, such as foaming and emulsifying abilities. The measurement of the content of free-SH groups located on the surface of okara proteins was used to provide further insights into the ability of sonication to cause changes in the protein tertiary structure. 

**Figure 3:**
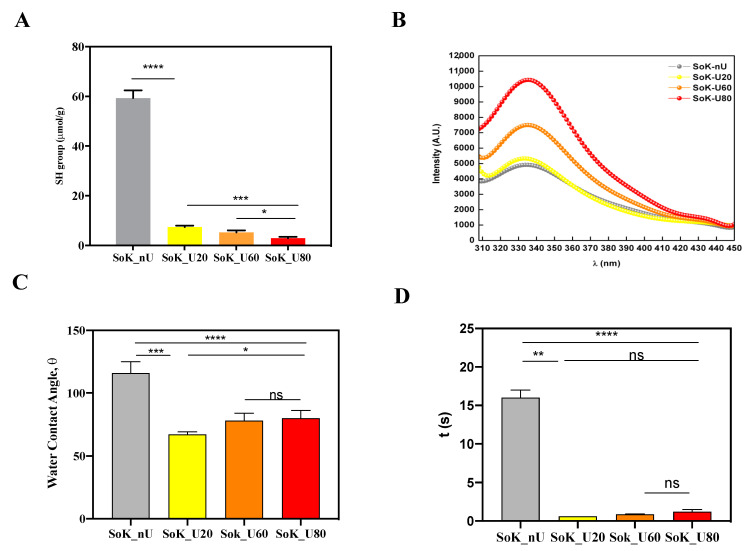
Tertiary structure analysis. (**A**) Free SH group determination. (**B**) Intrinsic fluorescence signal detection. (**C**) Water contact angle. (**D**) Time (s) of water absorption of proteins extracted with and without ultrasound and temperature treatments. SoK_nU (untreated), Sok_U80 (ultrasonicated at 80 °C), Sok_U60 (ultrasonicated at 60 °C), and Sok_U20 (ultrasonicated at 20 °C). Statistical analysis was performed by one-way ANOVA; (*) *p* < 0.5, (**) *p* < 0.01, (***) *p* < 0.001, and (****) *p* < 0.0001; ns: not significant. The data are represented as the means ± s.d. of three independent experiments.

Figure 3A shows significant reductions in free SH content of the extracted proteins after sonication. In detail, the free SH contents of SoK_nU was 59.4 ± 3.1 μmol/g and the ultrasonication at 20 °C led to a reduction of the free SH content up to 7.4 ± 0.6 μmol/g. These findings clearly indicate that the ultrasonication determines a significant effect on the structure of the protein with a reduction of the exposition of the hydrophobic amino acids containing thiol groups, probably due to the formation of intermolecular disulfide bonds (S-S), which modulates the folding of the extracted proteins. Moreover, the findings suggest that an increment of the temperature induced an additional decrease of the free SH content by 28.4% and 60.8% for SoK_U60 (5.3 ± 0.8 μmol/g) and SoK_U80 (2.9 ± 0.6 μmol/g), respectively, versus SoK_U20. These data underline the effects of two different factors: the ultrasonication process and the increasing temperature that induces extensive modifications of the protein structures. This phenomenon may be possibly due to the generation of radical species during the sonication process, which may oxidize susceptible functional groups such the thiol group, leading to the formation of intermolecular disulfide bonds (S-S). Indeed, the thermolysis induced by cavitation may produce hydroxyl radicals and hydrogen atoms that can induce the formation of radical species [35]. However, other researchers have reported that the sonication can increase the free SH content of egg and soy proteins [36]. The protein type, the solutions, and the processing conditions may produce these different outcomes.

### 3.5. Intrinsic Fluorescence

More information regarding the effect of ultrasound at different temperatures on the protein structural changes was obtained by applying intrinsic fluorescence spectroscopy, a technique that can be used to monitor alterations in protein tertiary structure due to the sensitivity of the protein amino acid residues to the polarity of the microenvironment [37]. Since the intrinsic fluorescence is mainly due to the presence of tryptophan (Trp) and tyrosine (Tyr) residues, which have strong fluorescence quantum yield, after excitation at 280 nm, the fluorescence spectrum of each sample was recorded in the wavelength range 310–450 nm. An improvement of fluorescence intensity was detected as a function of the temperature reached during the ultrasound-assisted protein extraction from okara (Figure 3B). The SoK_U20 had a significant 5.7% increase of fluorescence intensities compared to the untreated sample (SoK_nU) whereas, the fluorescent intensity of SoK_U60 and SoK_U80 was increased by 43.5% and 103.2%, respectively, compared to SoK_U20. The increased fluorescence intensity is correlated to an increase in the number of exposed Trp residues. Similar trends have been observed also in soybean and chicken plasma proteins submitted to ultrasound extraction [21,36]. In particular, a recent paper has shown that in soybean all sonication conditions induce a significant 13–41% increase of the fluorescence intensities compared to the untreated sample [21]. 

The changes of protein tertiary structure may be determined by monitoring fluorescence intensity at the maximum wavelength (λ_max_) [38]. In these experiments, SoK_U60 reached the λ_max_ at 332 nm, whereas all the other samples displayed a λ_max_ at 340 nm. Trp resides can be classified into three types based on their different λ_max_ values [39]: (i) buried Trp residue at λ_max_ between 330 and 332 nm, (ii) exposed Trp with limited water contact at λ_max_ between 340 and 342 nm, and (iii) exposed Trp residue at λ_max_ between 350 and 353 nm. Hence, Trp is exposed with limited water contact in SoK_nU, SoK_U20, and SoK_U80, whereas it is buried in SoK_U60. The slight shift in λ_max_ from 332 nm to 340 nm of SoK_U60 indicates a different behavior of this sample, since the Trp residues have shifted from being exposed with limited water contact to be buried.

### 3.6. Protein Hydrophobicity After Ultrasound Reatments

In order to assess the variation of the hydrophobicity, the water contact angles of the samples were measured (Figure 3C,D). Briefly, sample powders were deposited on glass slides and a drop of water was produced and fell on the surface. As shown in the videos (see Supplementary Materials), the proteins extracted with ultrasound coupled to the temperature gradient showed an improved ability to absorb the water drop in comparison with the untreated sample (SoK_nU). Moreover, SoK_nU has a slower ability to absorb water and an improved ability to swell. On the contrary, SoK_U20, SoK_U60, and SoK_U80 absorbed the water drop faster without swelling (Figure 3C,D), suggesting an improvement of their wettability. Precisely, the water contact angles (θ) of SoK_U20, SoK_U60, and SoK_U80 were the 42.7 ± 2.1%, 67.2 ± 4.5% and 68.9 ± 5.2%, respectively, versus SoK_nU (Figure 3C). Therefore, all values were smaller than that of the untreated sample, but slight improvements were detected increasing the temperature of the ultrasound treatment. Notably, the water drop was absorbed in 16 s by SoK_nU, whereas in 0.6 s, 0.9 s, and 1.2 s by SoK_U20, SoK_U60, and SoK_ U80, respectively (Figure 3D).

### 3.7. Morphological Analysis

**Figure 4:**
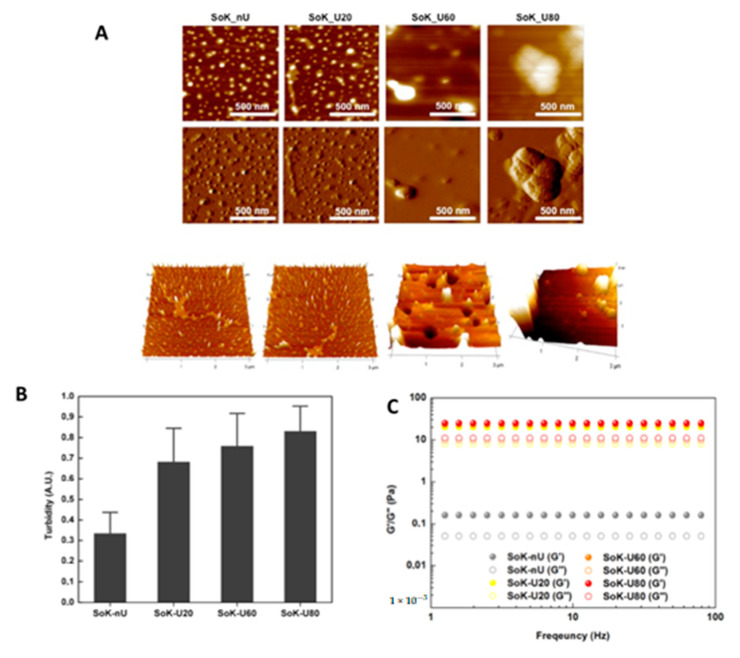
(**A**) Morphological organization of extracted proteins. (**B**) Turbidity of okara proteins measured at 405 nm. (**C**) Frequency-depended oscillatory rheology (1–100 Hz) of extracted proteins at fixed strain 0.1%. SoK_nU (untreated), Sok_U80 (ultrasonicated at 80 °C), Sok_U60 (ultrasonicated at 60 °C), and Sok_U20 (ultrasonicated at 20 °C).

To investigate the effect of the ultrasound and heat treatments on the extracted proteins, the morphologies of the samples were studied by AFM (Figure 4A). Well-defined single round structures were observed in SoK_nU alone, with an average height of 1.6 ± 0.9 nm (Figure S2) and no obvious changes of aggregates were found in SoK_U20 (2.0 ± 1.7 nm). Instead, SoK_U60 showed unevenly distributed aggregated structures, with a slightly increased height compared to SoK_nU and SoK_U20, namely, 5.6 ± 1.7 nm. Conversely, in sample SoK_U80, a significant morphological transition from well-defined single round structures to highly aggregated structures was observed. The size of the structures was increased compared to that of SoK_nU alone and SoK_U20, with a height of 47 ± 10.9 nm, indicating that the ultrasound coupled with an 80 °C temperature strongly contributed to the formation of these large aggregates. 

These data are in good agreement with those obtained through the turbidity assay (Figure 4B), where an increase in turbidity values was observed following heating of the samples, suggesting that the higher temperature led to a significant increase in the size of the protein aggregates. 

In addition, rheological experiments were performed to investigate the storage (G’) and loss (G’’) moduli in the function of angular frequency (1–100 Hz). All samples exhibited a G’/G’’ profile almost unchanged along the tested frequency range (Figure 4C). However, the ultrasound treated samples displayed higher G’ values (≈25 Pa) compared to the untreated sample (SoK_nU, G’ = 0.2 Pa); this behavior may indicate different networks of nano/microstructures inside the extracted protein, thus potentially influencing their mechanical properties.

### 3.8. Protein Solubility, Water Binding Capacity (WBC), and Viscosity of Extracted Proteins

**Figure 5:**
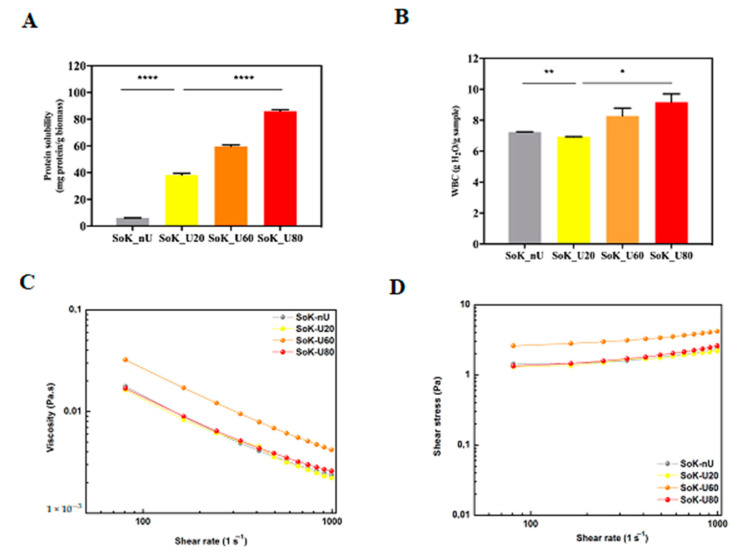
Solubility and rheological properties. (**A**) Protein solubility. (**B**) Water binding capacity (WBC). (**C**) Viscosity measurements at increasing shear rate. (**D**) Shear stress (σ) measurements at increasing shear rate of extracted proteins confirmed the non-Newtonian shear-thinning behavior of untreated and sonicated proteins. SoK_nU (untreated), Sok_U80 (ultrasonicated at 80 °C), Sok_U60 (ultrasonicated at 60 °C), and Sok_U20 (ultrasonicated at 20 °C). Statistical analysis was performed by one-way ANOVA; (*) *p* < 0.5, (**) *p* < 0.01, and (****) *p* < 0.0001. The data are represented as the means ± s.d. of three independent experiments.

The ultrasonication process affected also the protein solubility, which represents a good index of protein functionality [39]. This feature reflects protein denaturation and aggregation, which modulate many important functional properties, such as emulsification, solubility, gelation, and viscosity [40]. The solubility of untreated and treated samples by ultrasound is shown in Figure 5A. The protein solubility of SoK_U20 is 6.5-fold higher (38.2 ± 1.3 mg protein/g biomass SoK_U20) than the untreated sample (5.9 ± 0.1 mg protein/g biomass). In addition, the temperature during the ultrasound process proportionally increases the protein solubility that for SoK_U60 (59.6 ± 1.2 mg protein/g biomass) and SoK_U80 (86.1 ± 1.1 mg protein/g biomass) were greater by 1.5 and 2.3 folds, respectively, than that of SoK_U20 (Figure 5A). This evidence is supported by the fact the during ultrasonication the cavitation bubbles induce the unfolding of proteins causing an increased exposure of hydrophilic amino acid residues towards water thus contributing to the formation of soluble protein aggregates [41]. In addition, the increase of peptides within the samples may also contribute to the improvement of solubility.

An important property of proteins is the ability to interact with water, which influences their propensity to form gels, to dissolve, to swell, and to act as stabilizer in emulsions [42]. The measurement of their WBC is a conventional way to describe the interaction of proteins with water. Therefore, in order to evaluate the effect on WBC, dedicated experiments were carried out (Figure 5B). The WBC of the proteins obtained with the classical extraction method (SoK_nU) was 7.2 ± 0.01 g H_2_O/g protein (Figure 5B), in line with a previous study, which compared the WBC of soybean, pea, and lupin isolated proteins, reporting that the specific WBC of soybean proteins is 9.2 g H_2_O/g protein respectively [43]. Moreover, the ultrasonication at 20 °C (SoK_U20) did not produce a significant effect on the WBC (7.0 ± 0.01 g H_2_O/g protein). On the contrary, a significant variation of WBC was observed after ultrasound extraction at 60 and 80 °C: the WBC’s of SoK_U60 and SoK_U80 were 18 ± 0.5% and 31.4 ± 0.8% higher than SoK_U20, respectively (Figure 5B). 

In light of these observations, rheology was employed to evaluate the viscous properties: All samples displayed a non-Newtonian shear-thinning behavior with a decrease of viscosity that was concomitant with the shear-rate increase (Figure 5C). Even if the SoK_U60 showed an increased viscosity (0.032 Pa.s) in respect to samples SoK_nU (0.017 Pa.s), SoK_U20 (0.016 Pa.s), and SoK_U80 (0.016 Pa.s), all samples had negligible differences at higher shear rate values (700–1000 s^−1^). The non-Newtonian shear-thinning behavior of all samples was also confirmed by assessing the shear stress (σ) trend alongside shear-rate increments (Figure 5D).

### 3.9. In Vitro Antioxidant Activity Assayed by DPPH

**Figure 6:**
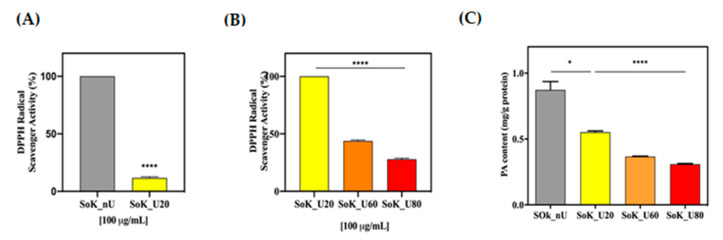
(**A**,**B**) Antioxidant evaluation of SoK_nU, SoK_U20, SoK_U60, and SoK_U80 by DPPH assay; (**C**) PA content determination. SoK_nU (untreated), Sok_U80 (ultrasonicated at 80 °C), Sok_U60 (ultrasonicated at 60 °C), and Sok_U20 (ultrasonicated at 20 °C). Statistical analysis was performed by one-way ANOVA (*) *p* < 0.5, (****) *p* < 0.0001. The data are represented as the means ± s.d. of three independent experiments.

The DPPH radical scavenging assay is one of the most commonly used single electron transfer (SET) based antioxidant procedures. Each sample was tested at the concentration of 0.1 mg/Ml. A comparison of the result of the untreated sample with that of the sample treated with ultrasound at room temperature shows that this treatment increases the ability of the sample to scavenge the DPPH radical: in fact, Sok_U20 diminished the DPPH radicals by 90 ± 5.6% compared to SoK_nU (*p* < 0.0001) (Figure 6A). In addition, further improvements of the antioxidant capacity are induced by the thermal treatments, since SoK_U60 and SoK_U80 reduced the DPPH radical by 56.3 ± 0.6% and 72.2 ± 0.3%, respectively (*p* < 0.0001, Figure 6B). The increased antioxidant capacity may be due to the exposure of hidden amino acid residues and side chains with antioxidant capacities (which are usually hidden within the three-dimensional structure of protein molecules). However, it is also important to underline that the peptidomic analysis had already shown that a higher temperature during the ultrasound treatment has the consequence of a relevant increment of the presence of short peptides that may have additional roles in the detected antioxidant activity. Moreover, previous studies carried out on different food matrices (soybean included) have demonstrated that an improved antioxidant ability might be attributed to the formation of short-chain peptides induced by the ultrasound treatment [21].

### 3.10. Phytic Acid Reduction by Ultrasound Treatment

Since literature indicates that ultrasounds may be successfully applied to reduce the anti-nutritional factor phytic acid [44], it was decided to investigate also this aspect. By normalizing the content of phytic acid in respect to the protein content of each sample, our finding showed that the ultrasonication coupled to temperatures simultaneously reduced the phytic acid proteins interactions compared to the raw sample as reported in (Figure 6C). In detail, a reduction of phytic acid content by 37.0 ± 1.0% was observed in SoK_U20 (0.5 ± 0.01 mg/g of protein) versus SoK_nU (0.9 ± 0.06 mg/g of protein) (*p* < 0.05, Figure 6C). This may be explained considering that the acoustic effect of cavitation leads to a disruption of phytic acid, mainly localized in bran layer of soybean okara, by increasing the area and extraction rate of phytic acid into the solvent. Furthermore, the increase of temperature leads to an additional significant reduction of the phytic acid content compared to the ultrasound extraction performed at 20 °C. In fact, in SoK_U60 (0.36 ± 0.03 mg/g of protein) and SoK_U80 (0.30 ± 0.05 mg/g of protein), the phytic acid content decreased by 28 ± 3% and 40 ± 5%, respectively, versus Sok_U20 (0.5 ± 0.01 mg/g of protein) (*p* < 0.0001, Figure 6C).

## 4. Conclusions

The effect of ultrasound on the separation and extraction has been extensively studied: It is widely accepted that this process intensifies the extraction of valuable components from soybeans, leading to an overall improvement of protein yield. In this context, our investigation confirms that the ultrasound assisted extraction coupled to a gradient of temperature is also a useful strategy to improve the recovery of proteins from soy okara by-products. From an economical and environmental point of view, these findings contribute to fostering the soy okara protein extracted by ultrasonication processing as valuable and high-quality products for new applications, thus providing a sustainable way to solve the environmental criticism related to the huge quantities of okara produced annually.

An overall consideration of our results permits to conclude that the ultrasound procedure coupled to a temperature gradient modifies in a significant way the protein secondary and tertiary structures. In fact, a local reduction of α-helices structures and an improvement of beta-sheets and random coil conformations were observed depending on the applied temperature. In addition, a reduction of the free thiol groups and a different distribution of Trp were also detected within the protein samples. The AFM analysis demonstrated a significant morphological transition from well-defined single round structures to highly aggregated ones after the ultrasonication at the higher temperature, suggesting that these aggregates possessed more hydrophilic surfaces and more hydrophobic cores than the untreated sample. This feature was confirmed by measuring the water contact angle, whose results clearly indicated that untreated samples were more hydrophobic than the treated ones, a fact further confirmed by the slower ability of the untreated sample to absorb water drop than ultrasound extracted proteins. All these results were in agreement with the enhancement of the protein yields induced by the ultrasound treatments at different temperatures.

The improvement of protein yields may have a twofold explanation: (a) the cavitation phenomenon induced by ultrasound process enhances the disruption of intact cells leading to an increased extracellular release; (b) the improvement of protein solubility has the consequence of an increase of the recovery. Notably, in this ultrasound extraction, the improvements of protein solubility and water binding capacity appear to depend on the temperature in a similar way. On the contrary, rheological experiments do not support any variation of protein viscosity.

Finally, from a functional point of view, the improvement of peptides generation and the different amino acid exposition within the protein after the ultrasound process led to an increase of the antioxidant properties of the samples and to a reduction of their PA content.

## Figures and Tables

**Table 1:** Samples description.

Sample ID	T (°C)	Ultrasound	Treatment Time (min)	Energy Input (kJ/L)
SoK_nU	20	-	-	-
SoK_U20	20	4.0 kW	0.5	24
SoK_U60	60	4.0 kW	0.5	24
SoK_U80	80	4.0 kW	0.5	24

**Table 2:** Percentage of secondary structure composition of soybean okara proteins.

Secondary Structure	SoK_nU	SoK_U20	SoK_U60	SoK_U80
Helix (%)	32.4	26.7	11.3	1.1
Beta (%)	18.5	28.1	33.2	37.7
Turn (%)	12.5	12.5	12.5	12.5
Random (%)	37.6	38.2	39.5	42.0

## Data Availability

The data presented in this study are available in this article and Supplementary Materials.

## Supplementary Materials

The following are available online at https://www.mdpi.com/2304-8158/10/3/562/s1. Table S1: Protein identified in SoK_nU, SoK_U80, SoK_U60, SoK_U20 after tryptic digestion. Table S2: Peptide sequences identified in Sok_U80, Sok_U60, Sok_U20, respectively after ultrasound treatment. Figure S1: Experimental setup of ultrasound-assisted extraction. Figure S2: Total ion chromatograms (TICs) of (A) Sok_U80; (B) Sok_U60 and (C) Sok_U20, respectively; Videos 1, 2,3, and 4: wettability of SoK_nU, SoK_U20, SoK_U60, and SoK_U80, respectively.
