# Supplementary material for: Assessment of the Physicochemical and Conformational Changes of Ultrasound-Driven Proteins Extracted from Soybean Okara Byproduct
Source: Foods. 2021 Mar 8;10(3):562. doi: 10.3390/foods10030562 (PMC7998950; doi:10.3390/foods10030562)
Supplement: Supplementary file 1 [file foods-10-00562-s001.pdf]

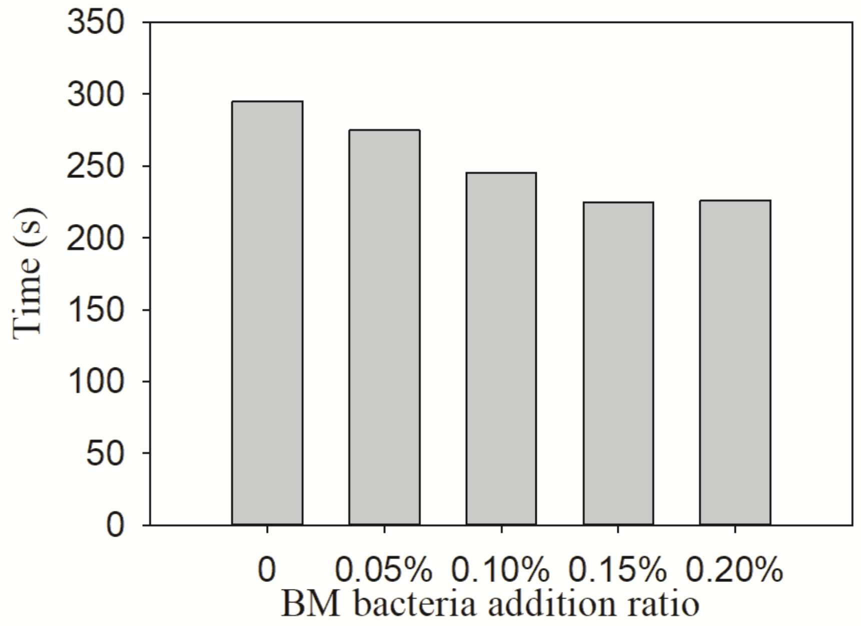

Figure.S1 Migration time of complex soil wetting front under different BM addition ratio. The percentage represents BM added mass ratio.

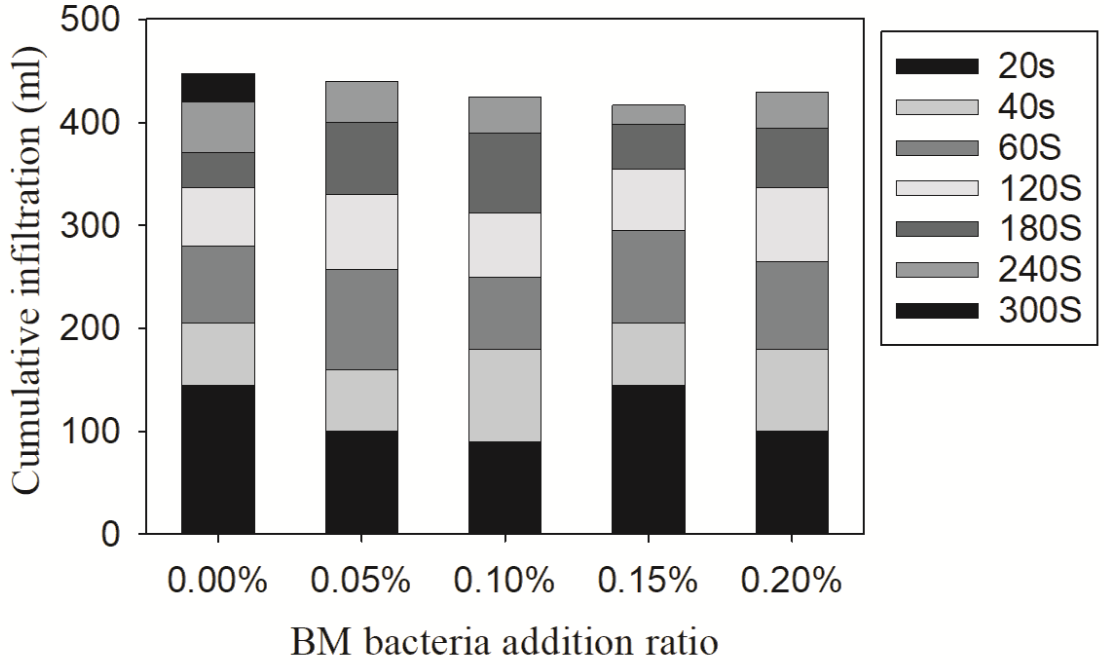

Figure.S2 Variation characteristics of cumulative infiltration of complex soil with different BM addition ratio within 5 min. The percentage represents BM added mass ratio.

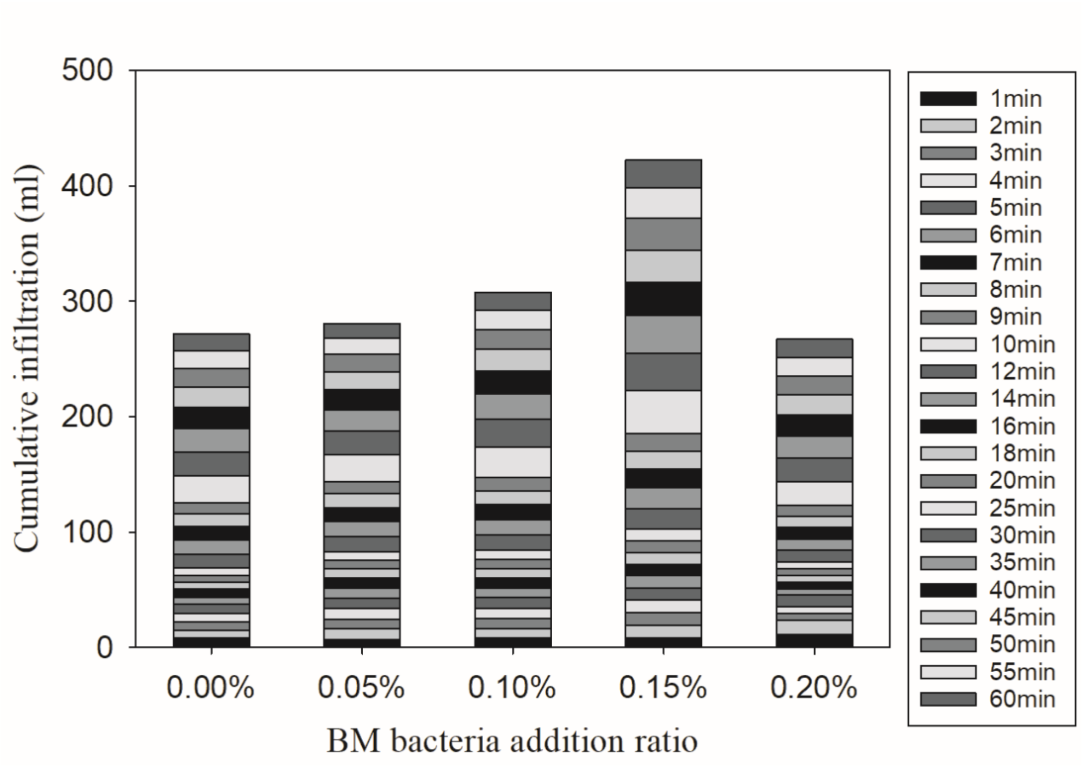

Figure.S3 Variation characteristics of cumulative infiltration of complex soil with different BM addition ratio under 60 min. The percentage represents BM added mass ratio.

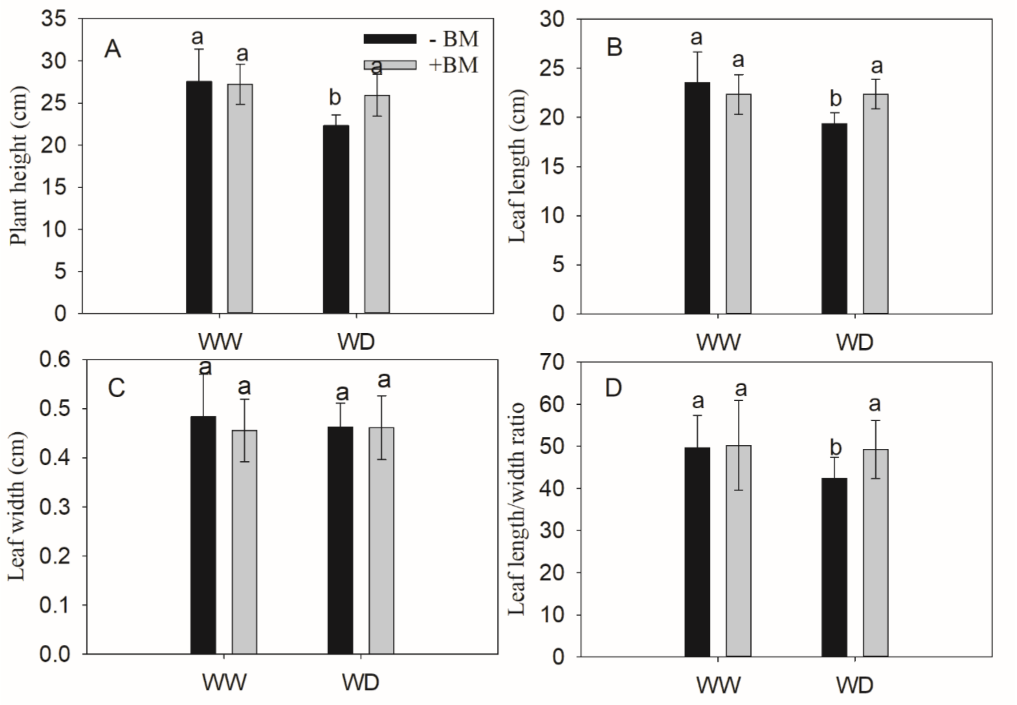

Figure. S4 Effect of BM application on plant height, leaf length and width of ryegrass under two water status. A, plant height; B, leaf length; C, Leaf width; D, leaf length/width ratio. WW and WD showed the different soil water content. The different letters indicate significant difference (p <0.05).
